# Research Progress on Natural Product Ingredients' Therapeutic Effects on Parkinson's Disease by Regulating Autophagy

**DOI:** 10.1155/2021/5538200

**Published:** 2021-04-24

**Authors:** Sicong Li, Xu Sun, Lei Bi, Yujia Tong, Xin Liu

**Affiliations:** ^1^School of Pharmacy, Peking University Health Science Centre, Beijing, China; ^2^Department of Pharmacy, The First Affiliated Hospital of Guangzhou University of Traditional Chinese Medicine, Guangzhou, China; ^3^School of Traditional Chinese Medicine, Beijing University of Traditional Chinese Medicine, Beijing 100029, China; ^4^Institute of Medical Information, Chinese Academy of Medical Sciences/Peking Union Medical College, Beijing, China

## Abstract

Parkinson's disease (PD) is a common neurodegenerative disease in middle-aged and older adults. Abnormal proteins such as *α*-synuclein are essential factors in PD's pathogenesis. Autophagy is the main participant in the clearance of abnormal proteins. The overactive or low function of autophagy leads to autophagy stress. Not only is it difficult to clear abnormal proteins but also it can cause damage to neurons. In this article, the effects of natural products ingredients, such as salidroside, paeoniflorin, curcumin, resveratrol, corynoxine, and baicalein, on regulating autophagy and protecting neurons were discussed in detail to provide a reference for the research and development of drugs for the treatment of PD.

## 1. Introduction

The prevalence of PD in the Chinese population over 65 years of age is 1.7% [1]. The main clinical features of PD are static tremor, bradykinesia, myotonia, and postural balance disorder. PD has two main pathological features: one is the degeneration and loss of dopaminergic neurons and other pigmented neurons in the substantia nigra compact area; and the other is eosinophilic inclusion bodies (Lewy bodies) in the cytoplasm of the remaining neurons. Overaccumulation of misfolded/aggregated proteins (e.g., mutant *α*-synuclein (A53T, A30P, and E46k)) or large accumulation of wild-type *α*-synuclein [2], as well as neurotoxins or mutant proteins (e.g., LRRK2 [3], DJ-1 [4], and PINK1 [5]), is responsible for the formation of Lewy bodies. Autophagy is an important mechanism for eukaryotic cells to clear damaged organelles, protein aggregates, and abnormal proteins using lysosomes. It can regulate the response to extracellular or intracellular pressure and signal, such as hypoxia, ischemia, growth factor deficiency, endoplasmic reticulum stress, and pathogen infection. Abnormal autophagy is a common cause of abnormal protein accumulation, such as *α*-synuclein and other proteins with aggregation tendency. Dopamine can react with *α*-synuclein in neurons and induce the posttranslational modification of *α*-synuclein. This process inhibits the fibrosis of *α*-synuclein protein and leads to the accumulation of *α*-synuclein protein in cells in the form of soluble toxicity, forming oligomer or oligoprotein, which eventually leads to injury or even death of neurons.

The etiology of PD is relatively complex, and there is still a lack of safe and efficient single treatment drugs, so we need to continue to strengthen the research on medicines to treat PD. Because of high efficiency and low toxicity, natural products have become the focus of current studies [6–9]. Natural products can eliminate misfolded *α*-synuclein by promoting autophagy. In this article, we reviewed the relevant researches on natural products, such as salidroside, astragaloside IV, paeoniflorin, curcumin, resveratrol, corynoxine, *β*-asarone, which eliminate the aggregation of abnormal protein by regulating autophagy to provide a reference for the development and researches of drugs for the treatment of PD.

## 2. Glycosides

### 2.1. Salidroside

Extracted from the dried roots and rhizomes of *Rhodiola rosea* L., salidroside has pharmacological effects of reducing pulmonary artery pressure [10], scavenging oxygen free radicals [11], promoting cell metabolism [12], and regulating cell autophagy [13]. mTOR is a receptor in the human body that regulates the autophagy signaling pathway and also plays an essential role in regulating cell growth, proliferation, and cell cycle. mTOR promotes neuronal survival, dendritic branching, and synaptic plasticity by phosphorylating the downstream substrate ribosomal 40S small subunit S6 protein kinase (70 kDa ribosomal protein S6 kinase, P70S6K). In vitro, salidroside pretreatment could activate *α*-synuclein clearance by promoting mTOR/p70S6K signaling pathway [10] and protect dopaminergic neurons from MPP+/MPTP damage by regulating NO pathway [13], ROS-NO-related mitochondrion pathway [14], and PI3K/AKT pathway [15]. Salidroside could reduce the necrosis and apoptosis of nerve cells by activating autophagy. Salidroside could make the LC3-II/LC3-I ratio rise and increase LC3-II and Bcl-2 expression and decrease Bax expression [16]. Pathologically, nearly 90% of the *α*-synuclein that made up the Lewy body was phosphorylated *α*-syn at Serine129 (p Ser129-*α*-syn). Salidroside could inhibit the increase of pSer129-*α*-syn level by improving the ubiquitin-proteasome system's function, thus alleviating 6-OHDA-induced neuronal injury [17]. Related to the occult formation of autophagosomes, PINK1 (PTEN-induced putative kinase 1) and Parkin have been research hotspots in the field of Parkinson's disease in recent years. Direct binding of PINK1 and Beclin 1 could promote the autophagosomal formation that was involved in mitochondrial dynamics, metabolism, and degradation of abnormal proteins [18]. PINK1 triggered mitochondrial autophagy by recruiting proteins, like Parkin, to the damaged mitochondrial surface [19]. In vivo, salidroside (50 mg/kg intragastrically administered for seven days) could significantly inhibit the expression of autophagy-pathway-related proteins in the nigra striatum of Parkinson's model mice (1-methyl-4-phenyl-1, 2, 3, 6-tetrahydropyridine model). The expression of LC-3*β* and Beclin 1 in substantia nigra striatum could be inhibited, while the expression levels of PINK1 and Parkin and the number of TH positive neurons increased [20].

In addition to inhibiting autophagy, salidroside could also protect dopaminergic neurons through antiapoptosis, antioxidation effect and inhibiting the inflammatory response. Salidroside (50 mg/kg·d, 100 mg/kg·d, and 200 mg/kg·d intragastrically administrated for 12 weeks) could dose-dependently inhibit neuronal apoptosis, decrease MDA content, increase SOD activity, and inhibit p-JNK3/JNK3 and caspase-3 protein expression in the hippocampus of rats after traumatic brain injury. These results indicated that salidroside inhibited oxidative-stress-mediated apoptosis of rat hippocampal neurons by inhibiting the JNK3/caspase-3 signaling pathway [21]. Salidroside could activate the TGF-*β*1/Smad3 signaling pathway in ischemic stroke model mice, inhibit Bax expression, and promote the expression of Bcl-2, TGF-*β*1, and p-Smad3, thereby reducing the damage of nerve function and playing a protective role on nerve cells [22]. In terms of anti-inflammation, salidroside (50 *μ*M) could not only indirectly reduce the production of NLRP3, pre-IL-1*β*, and pre-IL-18 by inhibiting the TLR4/MyD88/NF-*κ*B signaling pathway but also directly inhibit neuronal pyroptosis by inhibiting the TXNIP/NLRP3/caspase-1 signaling pathway [23, 24].

### 2.2. Astragaloside IV (ASI)

Astragaloside IV, extracted from the dried roots of *Astragalus propinquus* Schischkin, has pharmacological effects of regulating autophagy [19], anti-inflammation [25], anti-ischemic [26], antifibrosis [27], antitumor [28], and immunomodulation effects [29]. Before injecting MPTP, intraperitoneally injecting astragaloside IV (100 mg/kg, Q12 h) could promote mitochondrial autophagy and reduce the accumulation of damaged mitochondria and mitochondrial reactive oxygen species. Thus, it inhibited mice astrocyte senescence and degenerative changes of dopaminergic neurons, which significantly increased the contents of striatal dopamine and homovanillic acid and improved the movement of the mouse dysfunction [30, 31].

Astrocytes were bridges connecting neurons and blood vessels involved in neural development, neurotransmitter transmission, brain metabolism, and blood flow regulation [32]. Astrocyte apoptosis was also involved in the pathogenesis of neurodegenerative diseases like Parkinson's disease [33]. Astragaloside IV (6 mg/10 *μ*l) could inhibit the apoptosis of primary astrocytes induced by MPP+ and upregulate p-JNK, Bax/Bcl-2 ratio, and caspase-3 activity [34]. Astragaloside (1, 2, 5, 10, 20, and 50 *μ*g/ml) could promote the expression of tyrosine hydroxylase and dopamine transporter genes, Sonic hedgehog (Shh), Orphan nuclear hormone 1 (Nurr1), and Pituitary homeobox 3 (Ptx3) genes. Thus, it promoted the formation of neural stem cells into dopaminergic neurons [35].

Oxidative stress was one of the essential causes of mitochondrial dysfunction which participated in Parkinson's disease. Astragaloside (50, 100, and 200 µmol/l) could dose-dependently regulate p38 mitogen-activated protein kinase (MAPK) signaling pathway, downregulate the ratio of Bax/Bcl-2 to inhibit the expression of *α*-synuclein, increase the expression of tyrosine hydroxylase (THh) in cells, and protect SH-SY5Y cells from oxidative stress injury caused by H2O2 [36]. Astragaloside (30 mg/ml) could promote the JAK2/STAT3 signaling pathway to suppress the dopamine-induced Parkinson's model of SH-SY5Y cell apoptosis and oxidative stress injury [37]. Methionine sulfoxide reductase (Msr) had antioxidant effects and could repair proteins damaged by oxidative stress. Astragaloside (50 *μ*M) could upregulate Msr function through the SIRT1-FOXO3a signaling pathway, thereby regulating mitochondrial function and having an antioxidant effect [38]. Endoplasmic reticulum stress played a driving role in the apoptosis of dopaminergic neurons. Oxidative stress, glucose deficiency, hypoxia, and calcium imbalance could lead to the accumulation of unfolded or misfolded proteins in cells, leading to ER stress. Involved in apoptosis, the C/EBP homologous protein was a landmark protein of endoplasmic reticulum stress [39]. CHOP could directly promote autophagy-related genes ATG 10, ATG5, p62, and ATG 7 by binding to the cis-acting element GTGCAACC region of autophagy-related gene promoter [40]. The expression of long noncoding RNA (lincRNA-p21) could promote the ubiquitination of C/EBP homologous protein (CHOP) and accelerate the degradation of the protein. Astragaloside IV could inhibit the expression of lincRNA-p21 in Parkinson's disease model cells, thereby reducing the expression of CHOP protein and inhibiting the apoptosis of dopaminergic nerve cells [41]. Nucleotide binding and oligomerization domain-like (Nod) receptor family pyrin domain-containing 3 (NLRP3) inflammasome could promote the maturation and release of IL-1*β*, thus affecting the survival of dopaminergic neurons [42]. Inhibition of NLRP3 inflammasome activation could effectively improve neuroinflammation and dopaminergic neuron degeneration in PD. In terms of anti-inflammation, astragaloside IV significantly inhibited NF-*κ*B-mediated activation of NLRP3 inflammasome and Nrf2 in vitro and in vivo [43].

### 2.3. Paeoniflorin

Paeoniflorin, extracted from the dried roots of *Paeonia lactiflora* Pall. and *Paeonia suffruticosa* Andr., has regulating autophagy [37], anti-inflammatory [44], analgesic [45], and antitumor [46] effect.

Paeoniflorin could promote the binding of autophagosome and lysosome, significantly downregulate intracellular ROS level, and inhibit the oxidative damage in SH-SY5Y cells caused by H2O2 [47]. Paeoniflorin (50 *μ*mol/L) could upregulate the autophagy-lysosome and ubiquitin-proteasome pathways and promote the degradation of *α*-synuclein [48]. Paeoniflorin can also antagonize the damage of MPP+ and 6-OHDA on the autophagy-lysosome pathway [49, 50]. Sirtuins, especially SIRT1, were nicotinamide adenine dinucleotide dependent deacetylases that modulated the ubiquitin-proteasome pathway (UPP). UPP played an essential role in the normal function of neuronal synapses, synaptic protein turnover, plasticity, and long-term memory formation [51]. Paeoniflorin could improve cognitive impairment in mice with ischemia and hypoxia by activating SIRT1/NF-*κ*B signaling pathway. Paeoniflorin (5 mg/kg intragastrically administrated daily for 9 days) could inhibit the neuronal damage caused by rotenone in mice, reduce the formation of Lewy bodies, and inhibit the expression of *α*-synuclein [52]. Pathological activation of acid-sensitive ion channels was one of the causes of dopaminergic neuron degeneration in Parkinson's disease [53]. Paeoniflorin enhanced autophagy degradation of *α*-synuclein by blocking acid-sensitive ion channel 1A, thereby protecting dopamine neurons from 6-OHDA damage [54]. In terms of Alzheimer's disease, paeoniflorin protected SH-SY5Y cells from attack of okadaic acid by interfering with cal protease/Akt/GSK-3*β* related pathways and inhibiting the stress response of microtubule structural system [55].

However, low bioavailability and difficulty in crossing the blood-brain barrier limited paeoniflorin's therapeutic efficacy. In 2020, a kind of paeoniflorin polyamide nanocrystal and a new dosage form containing brain targeting ligand lactoferrin (Lf) and paeoniflorin (Pae) loaded in black phosphorus nanoplates were reported [56, 57]. These dosage forms could help paeoniflorin cross the blood-brain barrier and have good biocompatibility and biosafety.

## 3. Polyphenols

### 3.1. Curcumin

Curcumin, extracted from the dried roots of *Curcuma longa* L. and *Curcuma rcenyujin* Y., has pharmacological actions including anti-inflammatory [58], antioxidation [59], antitumor [60], antiapoptosis [61], and regulating autophagy [50] effects. In vitro, curcumin (40 *u*mol/L) could increase the expression of autophagy-related protein lysosome membrane protein 2 (ALAMP2A), microtubule-associated protein 1 light chain 3 (LC3-II), and nuclear plasma protein determination of nuclear transcription factor EB (TBBB) in Parkinson's disease model cells. Moreover, it could accelerate the transfer in the nuclei and the exercise of transcription TFEB function and promote autophagy-lysosome synthesis and autophagic clearance of *α*-syn [62, 63]. In vivo, intraperitoneally injecting curcumin could promote LC3-II protein expression in mice and inhibit the expression of P62, thus promoting autophagy. Intraperitoneally injecting curcumin could not only inhibit alpha Syn protein expression and DA neurons apoptosis in MPTP-induced Parkinson's disease model mouse (curcumin 80 mg/kg for 14 days) and improve the mouse movement disorder [10] but also improve the adult mice memory retrieval disorder induced by sevoflurane (curcumin 300 mg/(kg·d), for six days) [64]. In addition, curcumin could also activate autophagy by downregulating PI3K/Akt/mTOR signaling pathway, inhibit the damage of hippocampal neurons, and improve the learning and memory ability of rats [65].

In addition to promoting autophagy, curcumin's protective effect on nerve cells was also related to regulating MEK-ERK-CREB signaling pathways. In vitro 1 *u*M curcumin could significantly inhibit MPP+ induced JNK1, JNK2, upstream MKK4, and downstream c-Jun [66]. Curcumin could antagonize *α*-synuclein induced mitochondrial dynamic imbalance and corresponding functional damage by inhibiting the overactivation of ERK and the aggregation of mitochondrial mitotic protein DLP1 in mitochondria. At the concentration of 1 *μ*mol/L, curcumin could not only inhibit the activation of NADPH oxidase in microglia cells [67] and promote the expression of SIRT3 to eliminate microglia-derived ROS [68] but also inhibit the apoptosis of dopaminergic neurons by activating the IGF-1/Akt/FoxO3a signaling pathway [69] and PI3K/AKT/Bcl-2 signaling pathway [70].

### 3.2. Resveratrol

Resveratrol, extracted from grape, peanut, *Morus alba* L., and *Reynoutria japonica* Houtt., has anti-inflammatory [71], antioxidation [72], regulating autophagy [67], anticonvulsant [73], anti-ischemic [74], and antitumor [75] effects. Resveratrol played a neuroprotective role by activating SIRT1, promoting mitochondrial autophagy, and improving mitochondrial dysfunction [76]. SIRT1 regulated mitochondrial function and inhibited oxidative stress by maintaining the deacetylation state of peroxisome proliferator-activated receptor-*γ* coactivator 1*α* (PGC-1*α*), thus maintaining the constant level of PGC-1*α*. Resveratrol (20 mg/kg, intragastrically administered for 14 days) could protect dopaminergic neurons from the destruction of MPTP by activating the SIRT1/PGC-1*α*/autophagy pathway [77]. Moreover, it could improve the 6-OHDA-induced Parkinson's disease in mice by activating phosphatidylinositol 3 kinase (PI3K)/protein kinase (Akt)/glycogen synthase kinase 3*β* (GSK-3*β*) signaling pathway [78]. AMP-activated protein kinase (AMPK) controlled the metabolic balance in vivo by regulating autophagy and protein degradation and maintained the balance of cell energy supply and demand by affecting multiple links of cell glucose and fat metabolisms. Resveratrol (100 mg/kg) could inhibit mitochondrial damage and apoptosis of dopaminergic neurons in the substantia nigra of PD model mice induced by MPTP [79] and rotenone [80] by activating the SIRT1/AMPK signaling pathway after 33 days of intragastric administration. Resveratrol (50 mg/kg, intragastrically administered for nine weeks) could inhibit autophagy in rats with chronic cerebral ischemia by activating the PI3K/Akt/mTOR signaling pathway [81]. In vitro, 60 *μ*m/L resveratrol can upregulate the expression of PD-related genes PARKIN1, DJ-1, and PINK1 in zebrafish and inhibit the destruction of dopaminergic neurons induced by MPTP by regulating the Nrf2/ARE pathway [82]. As one of the antioxidative stress pathways in vivo, the NRF2/ARE pathway can regulate the REDOX level by regulating detoxification enzymes' transcriptional level.

Poor water solubility, unstable chemical properties, and low bioavailability limited resveratrol's efficacy. Resveratrol loaded in liposomes [83], polysorbate 80-coated poly(lactide) nanoparticles [83], and vitamin E loaded resveratrol nanoemulsion [84] exhibited better therapeutic effects than free resveratrol. In order to promote resveratrol to cross the blood-brain barrier, Li et al. [85] designed a series of pyridoxine-resveratrol hybrids as monoamine oxidase B inhibitors. Some of the compounds showed low cytotoxicity, good antioxidant activities, and high blood-brain barrier permeability.

## 4. Benzene

### 4.1. *β*-Asarone

Extracted from *Acorus tatarinowii*, *β*-asarone has anti-ischemic [86], antidepression [87], regulating autophagy [74], and antiepileptic [88] effects. Combination of *β*-asarone (15 mg/kg) and L-dopa (60 mg/kg) (intragastrically administrated twice a day for 30 days) could promote levodopa converted into the brain dopamine by increasing the permeability of the blood-brain barrier [89, 90]. Moreover, it could adjust the JNK/Bcl-2/Beclin 1 pathway and HSP70/MEF2D/Beclin 1 molecular chaperone-mediated autophagy pathway to reduce dopaminergic neurons injury induced by 6-OHDA in Parkinson's model rats [91, 92]. *β*-Asarone could reduce the dose of levodopa by increasing the content of tyrosine hydroxylase and decreasing the content of dopamine decarboxylase in the brain tissues [93]. Endoplasmic reticulum autophagy also played an important role in the pathogenesis of Parkinson's disease. Protein kinase RNA-like endoplasmic reticulum kinase (PERK) was in dopaminergic neurons in the substantia nigra striatum of patients with Parkinson's disease. *β*-asarone could regulate endoplasmic reticulum stress-autophagy by regulating the inositol-demanding enzyme 1/X-box binding protein 1 (IRE1/XBP1) pathway [94] and the PERK/CHOP/Bcl-2/Beclin 1 pathway [95]. In vivo and in vitro, *β*-asarone could inhibit the expression of long noncoding RNAs MALAT1 and *α*-synuclein to inhibit dopaminergic neuronal damage induced by MPP+ [96].

## 5. Alkaloids

### 5.1. Corynoxine

Corynoxine, extracted from *Uncaria rhynchophylla* (Miq.) Miq. ex Havil., has sedative [97], antiepileptic [98], and autophagy promoting [84] effects. Considered as a natural autophagy enhancer, corynoxine could induce autophagy in a concentration-dependent manner by regulating the Akt/mTOR pathway and promoting the clearance of wild-type and A53T-*α*-syn in SH-SY5Y cells and PC12 cells at concentrations of 6.25, 12.5, and 25 *μ*M. This effect could be inhibited by 3-MA or enhanced by chloroquine [99].

Mammalian target of rapamycin (mTOR) could regulate age-related degenerative diseases and destroy transient proteins in cytoplasm and core organelles by regulating the autophagy-lysosomal pathway (ALP) [27]. Found to be an inhibitor of mTOR, corynoxine (15 *μ*M) could effectively eliminate *α*-synuclein overexpression in N2A cells by inducing autophagy. This effect was related to the downregulation of the PI3K/Akt/mTOR signaling pathway and RPS6KB1/P70S6K (ribosomal protein S6 kinase, polypeptide (1)) and upregulation of MAP2K2/MEK2 (mitogen-activated protein kinase (2)) and PLK1 (polo-like kinase (1) functions) [100]. Intragastric administration of 10 mg/kg corynoxine for 12 weeks could improve movement disorders in rotenone-induced Parkinson's disease mice. The effect was not significantly different from that of rapamycin (10 mg/kg) [101].

## 6. Flavonoids

### 6.1. Baicalein

Baicalein, extracted from *Scutellaria baicalensis* Georgi., has anti-inflammatory [102], antioxidation [103], antitumor [104], and antiepileptic [98] effects. The therapeutic effect of baicalein on Parkinson's disease is related to inducing autophagy and regulating gene expression, antioxidation, and anti-inflammation effects.

Baicalein (5, 10, and 20 *µ*mol/L) could dose-dependently promote the formation of autophagosomes in PC12 neurons, inhibit the activity of mTOR, and promote the clearance of *α*-syn. The effects above could be inhibited by 3-methyladenine [105]. Baicalein (100 mg/kg, intragastrically administrated for 6 weeks) improved rotenone-induced behavioral deficits, dopaminergic neuron loss, autophagy dysfunction, and mitochondrial dysfunction in mice [106]. Baicalein (280 mg/kg intragastrically administered for 7 days) could significantly improve Parkinson's model mice's abnormal behaviors induced by MPTP, which could shorten the total rod-climbing time, prolong the rotational latency, and increase the vertical movement [107].

In terms of regulating gene expression, the LIMK1 gene could not only participate in regulating synaptic remodeling and cytoskeleton stability of hippocampal neurons but also affect neuron migration by regulating Cofilin [108]. The SNCA gene was responsible for encoding the *α* -syn protein, whose mutation, duplication, or triplet could lead to autosomal dominant PD [109]. GLRA1 gene was closely related to anxiety disorder, schizophrenia, and hyperstartle response [110]. By analyzing the gene coexpression network, the baicalein regulation of LIM kinase 1 (LIMK1), SNCA, GLRA1, and other genes might play a core role in the gene coexpression network [111]. In terms of anti-inflammation, baicalein (30 mg/kg intragastrically administered for 7 days) could inhibit the aggregation of *α*-synuclein, activation of inflammatory bodies, and production of Cathepsin B after MPP + injected into the substantia nigra of rats, as well as the upregulating tumor necrosis factor-*α* (TNF-*α*), interleukin-1*β* (IL-1*β*), and IL-6 in the substantia nigra and striatum of rats [112]. Neuroinflammation induced by inflammasomes was one of the pathogenic mechanisms of degeneration of dopaminergic neurons in the substantia nigra. Baicalein (100 mg/kg, 200 mg/kg, and 400 mg/kg, intragastrically administered for 4 weeks) could inhibit MPTP-induced mice nerve inflammation by inhibiting the NLRP3/caspase-1/GSDMD pathway [113].

In terms of antioxidation, baicalein could significantly reduce ROS and MDA levels in the brain and increase the activities of SOD and catalase (CAT). Injury of the ubiquitin-proteasome system was involved in the pathogenesis of Parkinson's disease. Baicalein (10 mM) might attenuate proteasomal-inhibitor-induced apoptosis in PC12 cells by inhibiting mitochondrial pathways and caspase-8 and Bid-dependent pathways, which might be associated with its inhibition of reactive oxygen generation and glutathione depletion. 25 *μ*M baicalein could protect dopaminergic neuroblastoma SH-SY5Y cells by inhibiting calcium homeostasis imbalance and mitochondrial damage caused by 6-OHDA+ ascorbic acid [114]. Moreover, baicalein could protect mitochondrial dysfunction by AMP-responsive element-binding protein (CREB) and glycogen synthase kinase-3*β* (GSK-3*β*) pathways [115].

## 7. Summary

Characterized by static tremor, bradykinesia, myotonia, and abnormal postural gait, Parkinson's disease (PD) is a common degenerative disease of central nervous system in middle-aged and senile persons. Degeneration loss of dopaminergic neurons in substantia nigra (DA) and formation of eosinophilic inclusion body (Lewy body) can be widely found in PD patients. Autophagy can degrade *α*-syn and inhibit *α*-syn deposition in Lewy body. At present, the treatments of PD are mostly supplying dopamine in the brain, and there is no effective method to protect dopaminergic neurons, so it can only delay the progress of disease but cannot fundamentally treat PD.

Natural products are potential drug choices for the treatment of Parkinson's disease due to their wide range of sources, multiple therapeutic targets, and easy access. Natural products can have various pharmacological actions that can have therapeutic effects on Parkinson's disease, including antioxidation, anti-inflammation, and antiapoptosis [116, 117] effects.

As is summarized in Table 1, salidroside, resveratrol, and baicalein can promote the clearance of *α*-syn and protect dopaminergic neurons by regulating the JNK/Bcl-2/Beclin 1, HSP70/MEF2D/Beclin 1, and IRE1/XBP1 pathway and autophagy-related signaling pathways such as PERK/CHOP/Bcl-2/Beclin 1 and PI3K/Akt/mTOR. Abnormal aggregation of *α*-syn is the key pathological feature of Parkinson's disease. The SNCA gene is responsible for encoding *α*-syn, and the abnormal expression of this gene will cause abnormal metabolism of neurons. Meanwhile, mutations of PD-related genes, such as PRKN, PINK1, and DJ-1, also affect the level of mitochondrial autophagy and normal physiological functions. Natural products like salidroside, resveratrol, and baicalein can regulate ALAMP2A, SNCA, PRKN, PINK1, and DJ-1 genes to protect autophagy inhibiting the apoptosis of dopaminergic neurons and promoting the elimination of *α*-syn. In addition to regulating autophagy, salidroside, astragaloside IV, paeoniflorin, resveratrol, and baicalein can also play an antioxidative and anti-inflammatory role in the treatment of Parkinson's disease.

At present, natural products are mostly used in the form of the compound for the treatment of Parkinson's disease and rarely used in the form of monomer. Compared with the compound decoction, the monomer component has the advantages of easy access and definite pharmacodynamic components, making it easier to be popularized and applied in clinical practice. Currently, studies on the efficacy of natural products are still in the stage of animal trials, and relevant researchers should continue to finish clinical trials on this basis to promote the continuous development of Parkinson's disease drugs.

## Figures and Tables

**Table 1:** Chemical structures, pharmacological actions, and related signal pathways of natural product ingredients included in this article.

	Chemical structure	Pharmacological action	Signal pathways
Salidroside	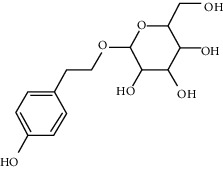	Regulating autophagy, antioxidation, anti-inflammation, and antiapoptosis	mTOR/p70S6K signaling pathway, JNK3/caspase-3 signaling pathway, ROS-NO-related mitochondrion pathway, and TGF-*β*1/Smad3 signaling pathway TLR4/MyD88/NF-*κ*B signaling pathway, TXNIP/NLRP3/caspase-1 signaling pathway, NO pathway, and PI3K/AKT pathway
Astragaloside IV	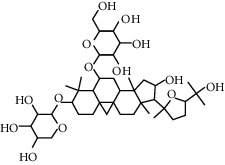	Regulating autophagy, antioxidation, anti-inflammation, and antiapoptosis	p38 MAPK signaling pathway, JNK3/caspase-3 signaling pathway JAK2/STAT3 signaling pathway, SIRT1-FOXO3a signaling pathway, and NF-*κ*B/NLRP3 signaling pathway
Paeoniflorin	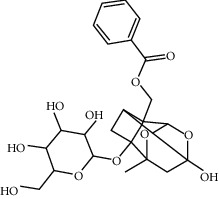	Regulating autophagy, antioxidation, anti-inflammation, and antiapoptosis	SIRT1/NF-*κ*B signaling pathway, PI-3K/Akt/GSK-3*β* signaling pathway
Curcumin	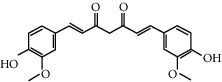	Regulating autophagy, antioxidation, anti-inflammation, and antiapoptosis	PI3K/Akt/mTOR signaling pathway, MEK-ERK-CREB signaling pathway, and IGF-1/Akt/ FOXO3a signaling pathway
Resveratrol	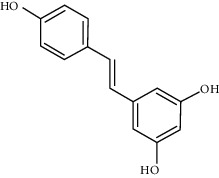	Regulating autophagy, antioxidation, anti-inflammation, and antiapoptosis	SIRT1/PGC-1*α* signaling pathway, PI3K/Akt/GSK-3*β* signaling pathway, SIRT1/AMPK signaling pathway, PI3K/Akt/mTOR signaling pathway, and Nrf2/ARE signaling pathway
*β*-Asarone	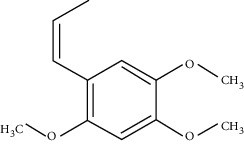	Regulating autophagy and endoplasmic reticulum stress, as well as antiapoptosis	JNK/Bcl-2/Beclin 1 pathway, IRE1/XBP1 pathway, and PERK/CHOP/Bcl-2/Beclin 1 pathway
Corynoxine	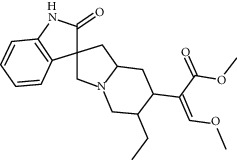	Regulating autophagy	Akt/mTOR pathway
Baicalein	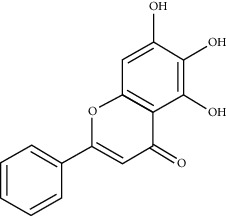	Regulating autophagy and gene expression, antioxidation, and antiapoptosis	NLRP3/caspase-1/GSDMD pathway, mitochondrial pathway, caspase-8 and Bid-dependent pathway, and CREB and glycogen synthase kinase-3*β* (GSK-3*β*) pathway

## Data Availability

The data used to support the findings of this study are included within the article.
